# In vivo deformation of anatomically pre-bent rods in thoracic adolescent idiopathic scoliosis

**DOI:** 10.1038/s41598-021-92187-y

**Published:** 2021-06-16

**Authors:** Hideki Sudo, Hiroyuki Tachi, Terufumi Kokabu, Katsuhisa Yamada, Akira Iwata, Tsutomu Endo, Masahiko Takahata, Yuichiro Abe, Norimasa Iwasaki

**Affiliations:** 1 Department of Orthopaedic Surgery, Hokkaido University Hospital, N15W7, Sapporo, Hokkaido 060-8638 Japan; 2 Department of Orthopaedic Surgery, Eniwa Hospital, Koganechuo 2-1-1, Eniwa, Hokkaido 061-1449 Japan; 3 Department of Orthopaedic Surgery, Faculty of Medicine, Graduate School of Medicine, Hokkaido University, N15W7, Sapporo, Hokkaido 060-8638 Japan

**Keywords:** Outcomes research, Musculoskeletal system

## Abstract

Some surgical strategies can maintain or restore thoracic kyphosis (TK); however, next-generation surgical schemes for adolescent idiopathic scoliosis (AIS) should consider anatomical corrections. A four-dimensional correction could be actively achieved by curving the rod. Thus, anatomically designed rods have been developed as notch-free, pre-bent rods for easier anatomical reconstruction. This study aimed to compare the initial curve corrections obtained using notch-free rods and manually bent, notched rods for the anatomical reconstruction of thoracic AIS. Two consecutive series of 60 patients who underwent anatomical posterior correction for main thoracic AIS curves were prospectively followed up. After multilevel facetectomy, except for the lowest instrumented segment, either notch-free or notched rods were used. Patient demographic data, radiographic measurements, and sagittal rod angles were analyzed within 1 week after surgery. Patients with notch-free rods had significantly higher postoperative TK than patients with notched rods (*P* < .001), but both groups achieved three-dimensional spinal corrections and significantly increased postoperative rates of patients with T6–T8 TK apex (*P* = .006 for notch-free rods and *P* = .008 for notched rods). The rod deformation angle at the concave side was significantly lower in the notch-free rods than in the notched rods (*P* < .001). The notch-free, pre-bent rod can maintain its curvature, leading to better correction or maintenance of TK after anatomical spinal correction surgery than the conventional notched rod. These results suggest the potential benefits of anatomically designed notch-free, pre-bent rods over conventional, manually bent rods.

## Introduction

The aim of adolescent idiopathic scoliosis (AIS) surgery is to correct the spinal deformity in the three planes. Since typical thoracic AIS shows a thoracic hypokyphosis leading to sagittal imbalance, correction of the thoracic kyphosis (TK) and the main thoracic (MT) curve is an important surgical goal^1–4^. To resolve further hypokyphosis of the thoracic spine secondary to pedicle screw instrumentations^1,5,6^, some posterior surgical strategies have been developed in which the TK is maintained or improved^4,7,8^.

Although these strategies can maintain or restore TK^4,7,8^, next-generation surgical schemes for patients with AIS should consider anatomical corrections^9^. Although the apex of the TK is located at T6–T8 in healthy humans^10^, the postoperative apex of the TK was almost identical with the apex of the preoperative MT scoliosis in some patients with kyphosis^10^. This non-anatomical TK is mainly generated by the rod-bending procedure, in which the configuration of the rods is approximated by rotation on the alignment of scoliosis before performing maneuvers in curve corrections^9^. Since the original shape of the rod is straight, intraoperative bending is needed to adapt the TK and lumbar lordosis. The initial shape of the spinal rod significantly affects spinal alignment^11–13^, and the rod-bending procedure highly depends on surgeons’ knowledge or experience, resulting in different configurations^9^. When the configuration does not adapt to the spinal deformity, it will lead to not only inadequate correction but also excess stress to the instrumentation and patient’s body. In addition, the manual bending procedure adds notches into the rod, which decreases its mechanical properties^14^.

**Figure 1:**
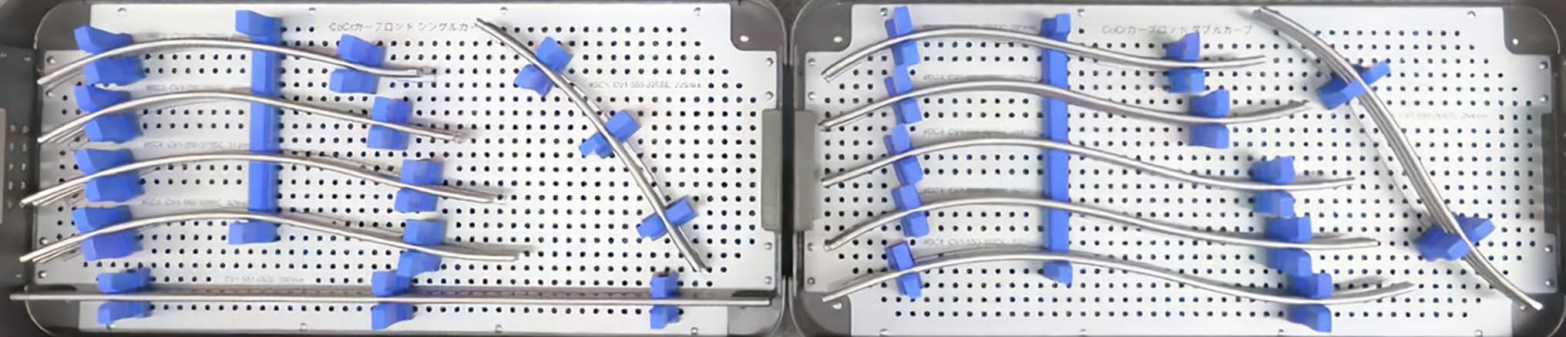
Anatomical notch-free, pre-bent rods for scoliosis surgery. Eleven types of rods can be utilized based on the length and curve types. The rod shapes were split up into two shapes: single curve and double curves. If the lowest instrumented vertebra (LIV) is L1 or above, two identical single-curve rods are used (left). If the LIV is L2 or L3, two identical double-curve rods are used.

From the spatiotemporal point of view, we recently documented that four-dimensional correction could be actively achieved by bending the rod under multilevel facetectomy^9^. In this surgical strategy, two rods were bent identically to guide postoperative anatomical TK without reference to the intraoperative coronal alignment of the deformity^9^. Thus, pre-bent configurations were intraoperatively derived from tracings of the rod shapes applied to the technique, and optimized rod configurations were identified to guide the anatomical reconstruction^15^. These results suggest that anatomically designed rods could be provided as notch-free, pre-bent rods^15^. The anatomical notch-free rods are created using custom-made rigid bending fixtures, and 11 types of cobalt chrome (CoCr) alloy rods can be utilized based on the curve types and its lengths. This medical device, which is the first in the world, has been approved by a national agency in Japan (Fig. 1).

The hypothesis underlying this study was that patients with notch-free, pre-bent rods would have a significantly higher postoperative TK than patients with conventional, manually bent, notched rods after anatomical spinal correction surgery. In addition, postoperative rod deformation, as a spring-back effect, would be reduced in patients with notch-free rods^14^. This study aimed to compare initial curve corrections obtained using notch-free rods and manually bent, notched rods for the surgical treatment of thoracic patients with AIS.

## Materials and methods

### Experimental design

The institutional review board of Hokkaido University Hospital approved the experiments, including any relevant details. All methods were performed in accordance with the relevant guidelines and regulations. Informed consent for this study and publication of identifying information/case study was obtained from all participants and/or their guardians/parents. Data from two consecutive series of patients who underwent posterior instrumentation surgery for Lenke 1 AIS curves were prospectively evaluated; all patients had a MT Cobb angle ≤ 90°. The exclusion criteria were syndromic, neuromuscular, and congenital scoliosis and Lenke 2–6 AIS curves. Manually bent, notched rods were used between 2014 and 2018, and notch-free rods were used between 2019 and 2020. No cases were lost to follow-up.

Standing radiographs were taken preoperatively and within 1 week after surgery, and Cobb measurements were obtained. Preoperative curve flexibility was measured using supine bending radiographs^4,9^. The end vertebrae levels were determined on preoperative radiographs and measured on subsequent radiographs to maintain consistency for statistical comparisons^9,16^. Coronal balance was evaluated by the lateral displacement of the C7 coronal plumb line from the center sacral vertical line (CSVL), and sagittal balance was measured as the sagittal vertical axis. A positive radiographic shoulder height (RSH) was defined as left shoulder up^17^. The angle of rotation of the thoracic apical vertebra was obtained on computed tomography (CT) images, as previously described^4,9,18^. Our internal studies of interrater and intrarater reliability have shown excellent kappa statistics for all continuous measures (0.90–0.98). The number of facetectomy levels was obtained, and screw density was evaluated as the number of screws per level instrumented^11^.

### Surgical technique

The instrumentation levels were primally decided to include the end vertebrae. The upper instrumented vertebra (UIV) was then selected based on the preoperative shoulder balance^19^ and anatomical TK; T3 was selected if RSH was >  − 5 to 0 mm, and T4 was selected if RSH was ≤  − 5 mm^9^. In case TK was < 20° and the upper-end vertebra was T5 or T6, the UIV selected was T4^9^. The last vertebra touching the CSVL was determined for the lowest instrumented vertebra (LIV) in cases of lumbar modifier A or B^19,20^. For lumbar modifier C, LIV was selected at L3.

Side-loading polyaxial pedicle screw instruments (USS II Polyaxial; DePuy Synthes, Raynham, MA, USA, for manually bent, notched rods or CVS spinal system; Robert Reid, Tokyo, Japan, for notch-free rods) were placed. All-level facetectomy was performed except for the lowest instrumented segment to avoid pseudoarthrosis at this site^9^.

For manually bent rods, two titanium alloy rods (φ 6.0 mm) were bent identically to guide the postoperative anatomical TK^9^. The apex was anticipated to be at T6–T8 for the postoperative TK^9^. The rod configurations were split into two shapes: single curve and double curves. In case the LIV was L1 or above, the single-curve rods were used^9^, and the thoracolumbar/lumbar region remained straight. When the LIV was L2 or L3, the double-curve rods were used^9^. For notch-free rods, CoCr alloy rods (φ 5.5 mm) were used. The rods also have two shapes, and each shape is provided by increments of 3 cm.

The rods were simultaneously rotated after connecting to the screw heads; in situ rod-bending procedure was not performed^4,9,11^. After the decortication of the laminae, local bones are grafted. Intraoperative monitoring using somatosensory/motor-evoked potentials was performed. None of the patients used brace.

### Analysis of rod configuration

**Figure 2:**
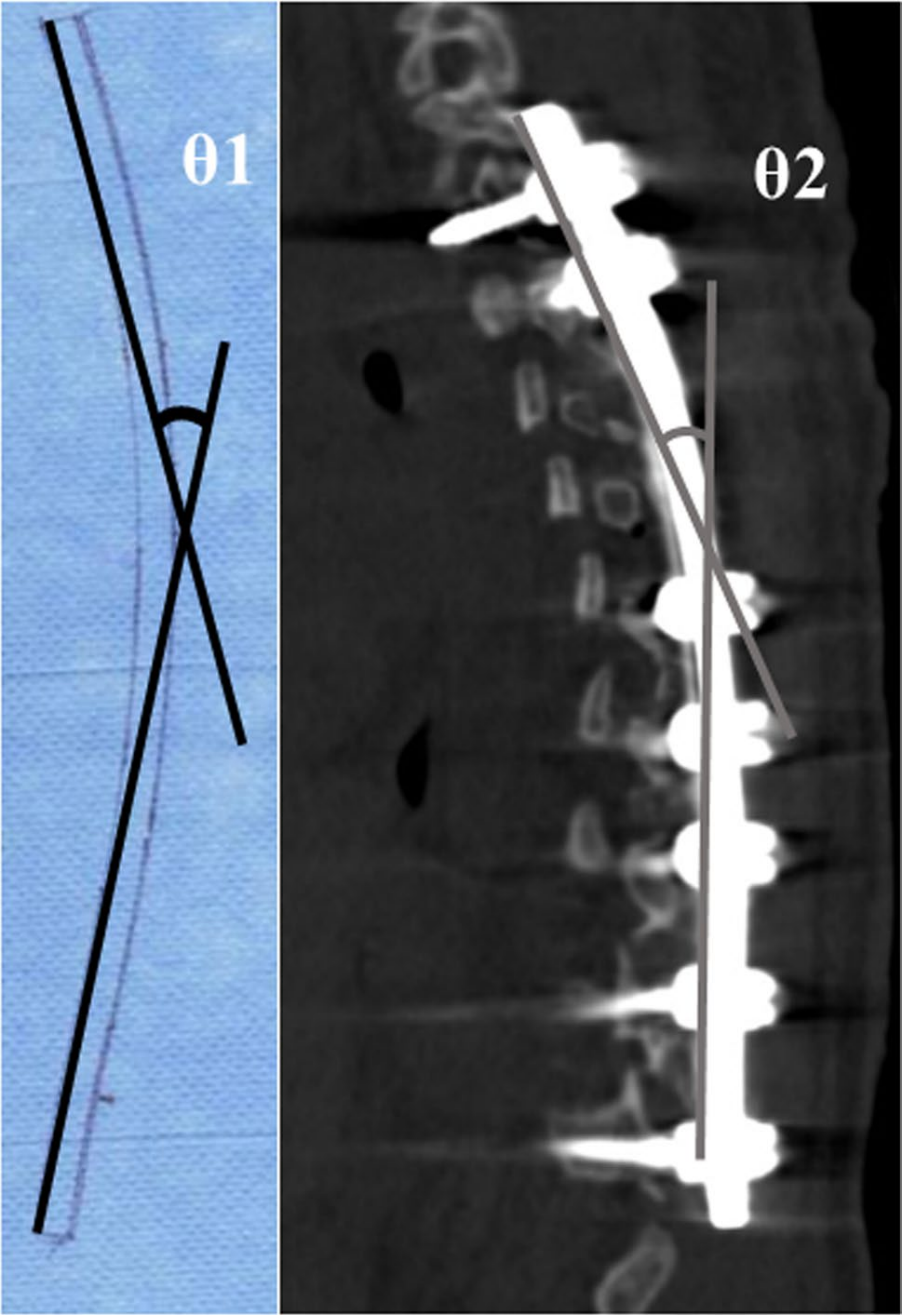
Rod angle before and after implantation. The angle between the proximal and distal tangential lines was measured. In case of notched rod, the surgeon traced the rod shapes on paper before implantation (θ1). The postoperative implant rod geometry (θ2) was obtained after the surgical operation using computed tomography.

For manually bent rods, rod shapes were traced on paper after intraoperative contouring of the rods^13,21^. The angle between the cranial and caudal tangential lines was measured before implantation (θ1) (Fig. 2)^13,21^. The postoperative rod angle was similarly measured (θ2) using reconstructed sagittal CT images^13^. If the rod had both thoracic and lumbar curvatures, the distal tangential line was set based on the inflection point^13^.

### Statistical analysis

All data are presented as means ± standard deviation and range. Comparisons of demographic data, radiographic quantitative variables, and rod angles were performed using a Mann–Whitney *U* test or a paired t-test. Chi-square tests were used to assess sex and lumbar modifier. All statistical analyses were conducted using JMP Pro version 14.0 statistical software (SAS Institute, Cary, NC, USA). A *P* value < 0.05 was considered statistically significant.

## Results

### Patient demographic data

There was no significant difference in age, sex, curve type, number of instrumented vertebrae, screw density, number of facetectomy levels, or operative time between the groups (Table 1).

**Table 1:** Patient demographic data.

	Notched rods	Notch-free rods	*P*
Number of patients (no.)	33	27	
Age at surgery (years)	15.2 ± 2.4 (12–20)	14.3 ± 2.5 (11–20)	0.173
Sex (no. and % of female)*	32 (97%)	24 (89%)	0.207
Risser sign (grade)	3.5 ± 1.5 (0–5)	3.7 ± 1.1 (0–5)	0.640
Cobb length (segments) (upper end to lower end vertebra)	8.3 ± 1.1 (7–10)	8.0 ± 1.2 (6–11)	0.183
Lumbar modifier (no. and %)*			
A	17 (52%)	17 (63%)	
B	6 (18%)	3 (11%)	
C	10 (30%)	7 (26%)	0.622
Number of instrumented vertebrae (segments)	10.7 ± 1.2 (9–13)	10.8 ± 1.2 (8–13)	0.866
Screw density at concave side (no. of screws/ levels instrumented)	0.9 ± 0.1 (0.7–1)	0.9 ± 0.1 (0.6–1)	0.479
Screw density at convex side (no. of screws/ levels instrumented)	0.8 ± 0.1 (0.5–1)	0.8 ± 0.1 (0.6–1)	0.261
Number of facetectomy levels (no.)	8.4 ± 1.3 (6–11)	8.7 ± 1.0 (7–10)	0.343
Operative time (min)	261.5 ± 47.6 (170–360)	251.6 ± 39.8 (190–322)	0.401

All data expressed as means ± SD and range. Mann–Whitney *U* test or Chi-square test*.

### Radiographic findings

**Figure 3:**
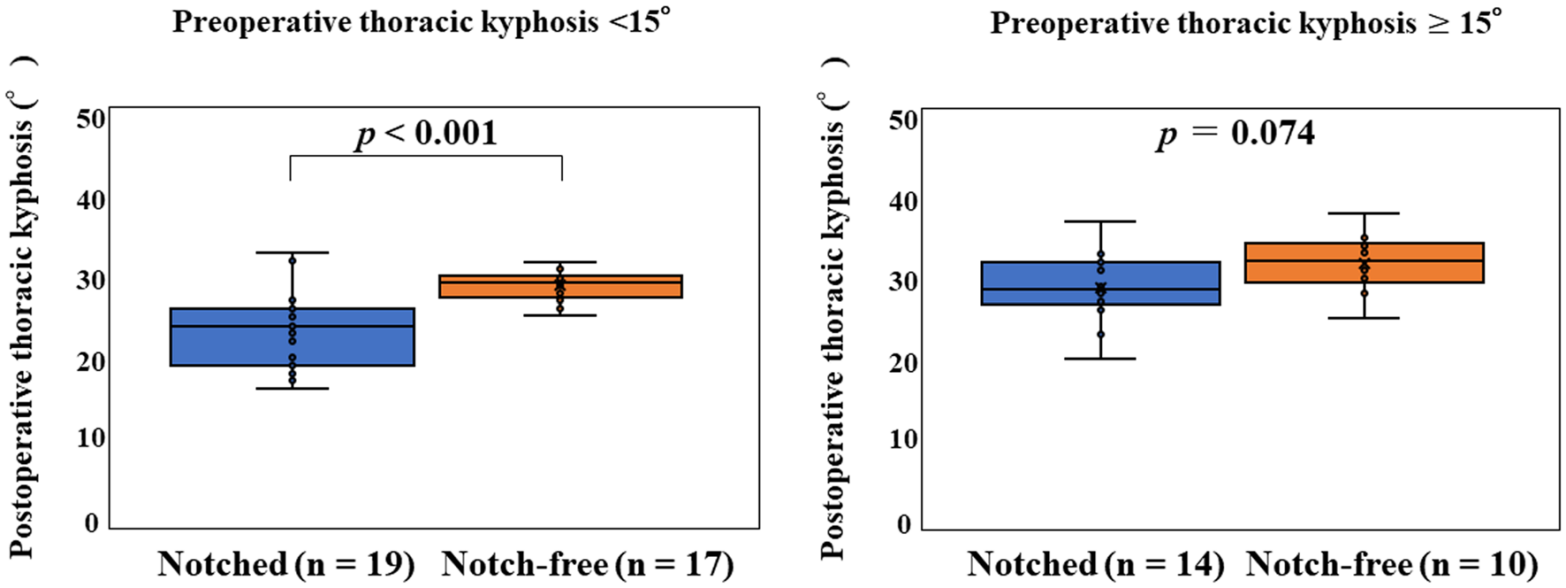
Postoperative thoracic kyphosis in patients with preoperative thoracic kyphosis < 15° and ≥ 15°.

There was no significant difference between the groups in terms of preoperative and postoperative coronal plane radiographic data (*P* > 0.05; Table 2). Sagittal plane analysis showed no significant difference between the groups in preoperative TK. However, postoperative TK and the change in TK were significantly higher in the notch-free rod group than in the notched rod group (*P* < 0.001 and *P* < 0.001, respectively). When the cohorts were divided into two groups based on preoperative TK, postoperative TK was significantly higher in the notch-free group than in the notched group in patients with preoperative TK < 15° (*P* < 0.001, Fig. 3), but both groups had similar baseline demographic data except for preoperative TK. There was no significant difference between the groups in terms of preoperative and postoperative lumbar lordosis, balance parameters, and translational data (*P* > 0.05).

**Table 2:** Radiographic parameters.

	Notched rods	Notch-free rods	*P*
**Coronal plane data**			
Preoperative main thoracic curve (°)	58.5 ± 8.0 (45–77)	55.9 ± 6.8 (45–75)	0.211
Preoperative flexibility of main thoracic curve (%)	67.4 ± 16.8 (23–91)	65.9 ± 17.0 (32–96)	0.505
Postoperative main thoracic curve (°)	8.6 ± 4.0 (1–20)	8.2 ± 4.4 (1–18)	0.182
Correction rate of main thoracic curve (%)	85.1 ± 7.1 (68–99)	85.5 ± 7.1 (71–98)	0.246
Preoperative thoracolumbar/lumbar curve (°)	26.2 ± 13.8 (1–46)	27.9 ± 10.2 (4–51)	0.953
Postoperative thoracolumbar/lumbar curve (°)	7.8 ± 6.5 (0–23)	6.9 ± 5.1 (0–20)	0.848
**Sagittal plane data**			
Preoperative thoracic kyphosis (T5 to T12) (°)	13.4 ± 6.5 (3–34)	14.2 ± 7.1 (2–31)	0.623
Postoperative thoracic kyphosis (T5 to T12) (°)	23.6 ± 5.3 (13–34)	30.0 ± 3.0 (25–38)	< 0.001
95% confidence interval	(21.8, 25.4)	(28.8, 30.9)	
Change in thoracic kyphosis (T5 to T12) (°)	10.1 ± 4.8 (0–20)	16.0 ± 5.6 (4–27)	<0.001
95% confidence interval	(8.7, 12.0)	(14.0, 18.2)	
Preoperative lumbar lordosis (L1 to S1) (°)	50.4 ± 9.7 (35–73)	47.0 ± 8.5 (30–65)	0.330
Postoperative lumbar lordosis (L1 to S1) (°)	47.3 ± 8.0 (29–62)	44.7 ± 7.5 (33–64)	0.097
**Balance parameters and translational data**			
Preoperative C7 translation from central sacral vertical line (mm)	11.7 ± 8.3 (0–27)	12.3 ± 7.2 (0–28)	0.929
Postoperative C7 translation from central sacral vertical line (mm)	10.2 ± 8.9 (0–26)	11.8 ± 7.0 (0–24)	0.532
Preoperative sagittal vertical axis (mm)	− 2.4 ± 19.8 (− 54 to 30)	− 7.7 ± 20.8 (− 50 to 28)	0.274
Postoperative sagittal vertical axis (mm)	− 4.4 ± 17.4 (− 37 to 40)	− 9.6 ± 11.7 (− 28 to 23)	0.136
Preoperative thoracic apical vertebral translation* (mm)	52.0 ± 12.9 (31–95)	50.5 ± 12.5 (27–77)	0.766
Postoperative thoracic apical vertebral translation* (mm)	7.8 ± 6.0 (0–27)	9.6 ± 4.6 (1–18)	0.148
Preoperative thoracolumbar/lumbar apical vertebral translation* (mm)	8.4 ± 7.9 (0–22)	9.3 ± 6.6 (0–23)	0.440
Postoperative thoracolumbar/lumbar apical vertebral translation* (mm)	6.2 ± 5.9 (0–25)	6.5 ± 5.9 (0–21)	0.928
**Shoulder balance data**			
Preoperative radiographic shoulder height (mm)	− 10.4 ± 8.1 (− 28 to 5)	− 9.4 ± 8.0 (− 23 to 9)	0.899
Postoperative radiographic shoulder height (mm)	7.4 ± 5.5 (0 to 18)	8.0 ± 5.0 (1 to 20)	0.956

All data expressed as means ± SD and range. Mann–Whitney *U* test.

*Distance between the geometric center of the apical vertebrae and the C7 plumb line or the center sacral vertical line.

**Figure 4:**
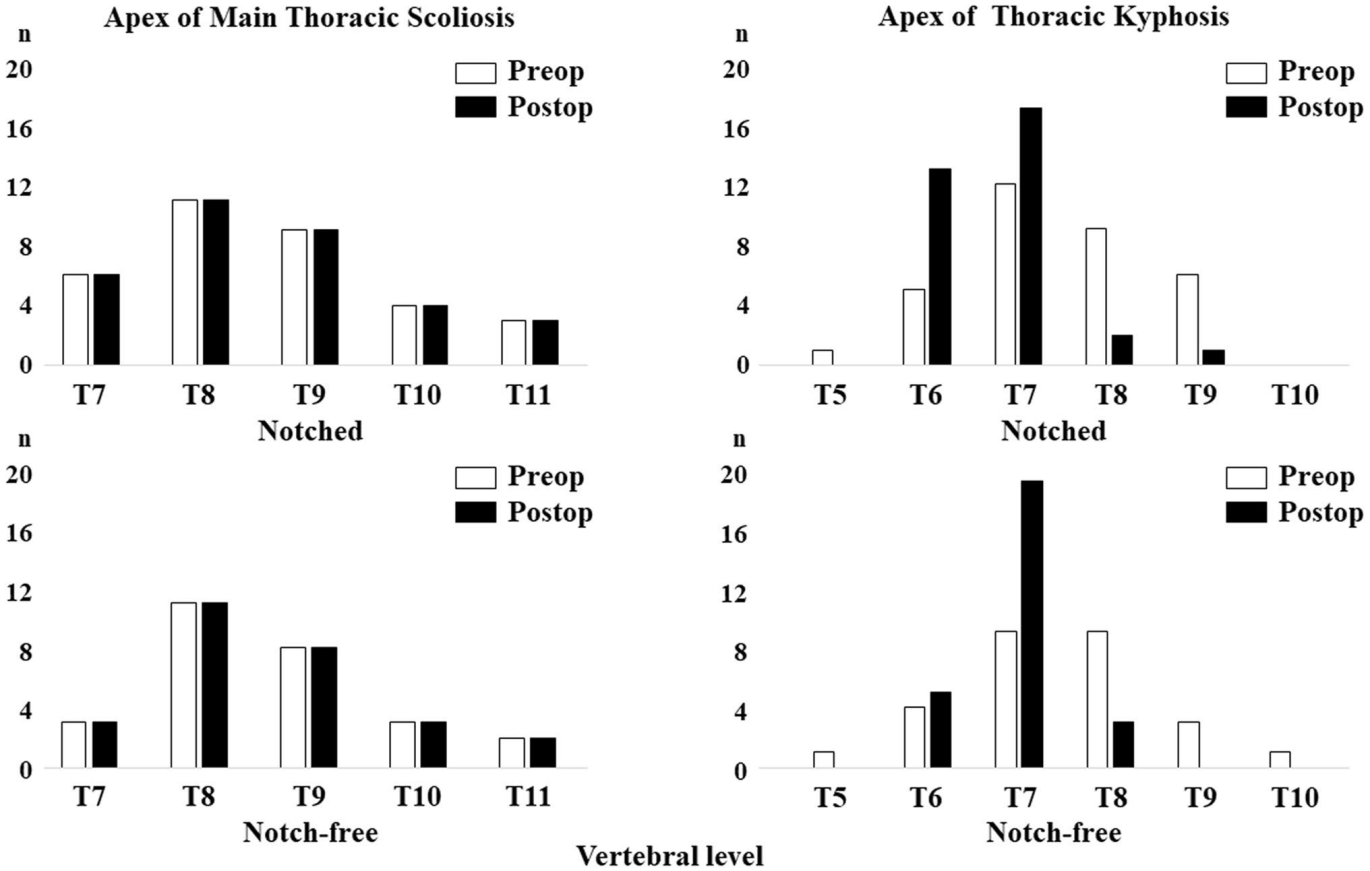
Apex of the main thoracic scoliosis and thoracic kyphosis.

The preoperative levels of the MT scoliosis and TK apices were different in 24 cases (73%) in the notched rod group and in 22 cases (81%) in the notch-free rod group, respectively (Fig. 4). The preoperative levels of the apex in MT scoliosis were the same postoperatively in both groups. The preoperative rates of patients with T6–T8 TK apex significantly increased from 76 to 97% postoperatively in the notched rod group (*P* = 0.008) and from 81 to 100% in the notch-free rod group (*P* = 0.006). There were no significant differences in the postoperative percentage of patients with T6–T8 TK apex between the groups (*P* = 0.271).

There was no significant difference between the groups in terms of preoperative and postoperative MT apical vertebral rotation angle (*P* > 0.05; Table 3).

**Table 3:** Vertebral rotation angle of main thoracic apical vertebra.

	Notched rods	Notch-free rods	*P*
Preoperative vertebral rotation (°)	17.9 ± 5.0 (8–32)	16.9 ± 5.1 (3–29)	0.134
Postoperative vertebral rotation (°)	11.5 ± 4.7 (0–24)	10.7 ± 4.8 (3–21)	0.111

All data expressed as means ± SD and range. Mann–Whitney *U* test.

### Implant rod angles of curvature

**Figure 5:**
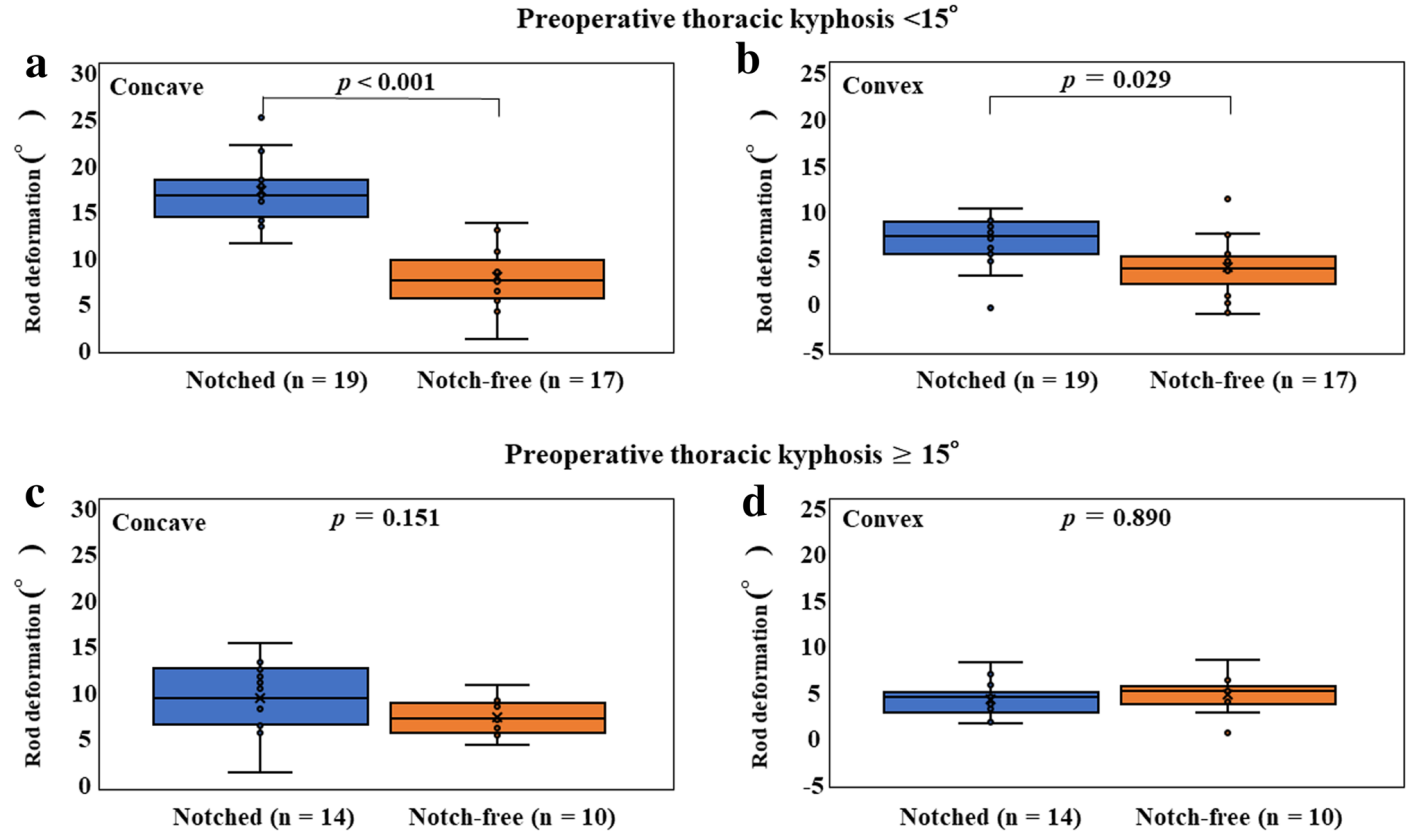
Rod deformation angles in patients with preoperative thoracic kyphosis < 15° **(a,b)** and ≥ 15° **(c,d)**.

The rod deformation angle at the concave side was significantly lower in the notch-free rod group than in the notched rod group (*P* < 0.001; Table 4). When the cohorts were divided into two groups based on preoperative TK, this significant difference was seen at both the concave and convex sides in patients with preoperative TK < 15° (*P* < 0.001 and *P* = 0.029, respectively), but not in patients with preoperative TK ≥ 15° (*P* = 0.151 and *P* = 0.890, respectively, Fig. 5).

**Table 4:** Implant rod angle of curvature at the concave and convex side of deformity.

	Notched rods	Notch-free rods	*P*
Preoperative rod angle (θ1) at concave side (°)	41.7 ± 4.4 (31.0–53.5)	40.2 ± 5.1 (33.6–46.0)	0.732
Preoperative rod angle (θ1) at convex side (°)	41.0 ± 4.9 (28.6–54.9)	40.2 ± 5.1 (33.6–46.0)	0.941
Postoperative rod angle (θ2) at concave side (°)	28.2 ± 5.4 (16.8–39.8)	33.1 ± 4.2 (26.4–40.1)	0.001
Postoperative rod angle (θ2) at convex side (°)	35.7 ± 5.2 (27.2–50.8)	36.6 ± 4.9 (27.2–42.8)	0.381
Rod deformation angle (Δθ) at concave side (°)	13.5 ± 5.2 (0.8–25.0)	7.1 ± 2.9 (0.3–13.3)	< 0.001
Rod deformation angle (Δθ) at convex side (°)	5.0 ± 2.6 (− 1.1 to 9.8)	3.7 ± 2.8 (− 1.8 to 10.9)	0.073

All data expressed as means ± SD and range. Mann–Whitney *U* test. Δθ was defined as the difference between θ1 and θ2 (θ1–θ2).

### Surgical complications

There were no surgical complications such as implant breakage or neurologic deficits in either group.

### Case presentation

**Figure 6:**
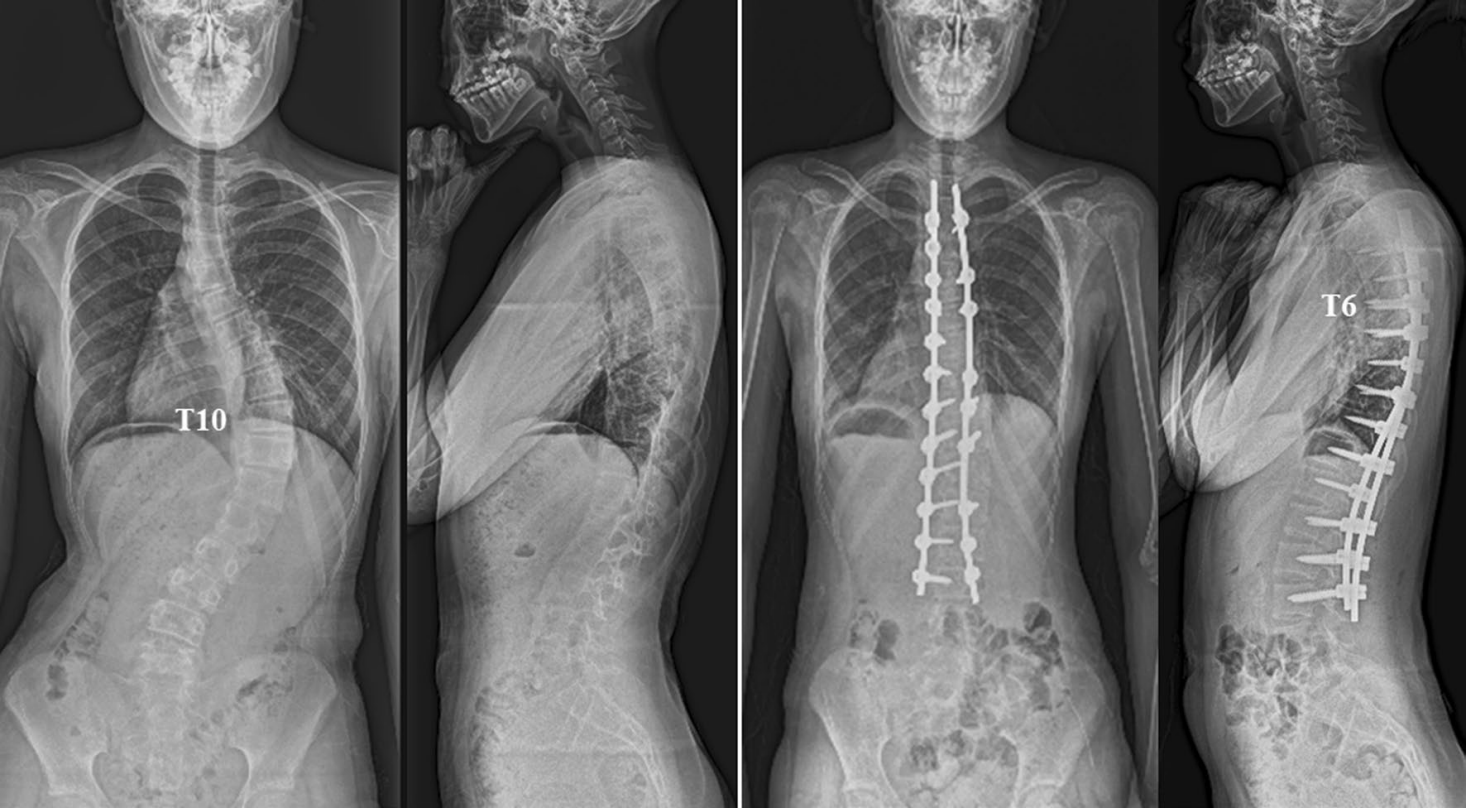
Anatomical spinal reconstruction using notch-free, pre-bent rods in a patient with Lenke 1A-thoracic adolescent idiopathic scoliosis.

A 14-year-old girl underwent anatomical spinal reconstruction using notch-free, pre-bent rods (Fig. 6); the MT Cobb angle was 63° (T7–L2), and the TK angle was 9° (T5–T12). The apex of the scoliosis was at T10. Postoperative radiographs obtained 1 week after surgery showed that the MT scoliosis and TK angles were 9° and 28°, respectively. The apex of the TK was at T6.

## Discussion

The most important findings of the present study were that patients with notch-free, pre-bent rods had a significantly higher postoperative TK than patients with conventional, manually bent, notched rods and that both groups achieved three-dimensional spinal corrections and significantly increased postoperative rates of patients with T6–T8 TK apex. The rod deformation angles were significantly lower in the notch-free rods than in the notched rods. These results suggest that the notch-free rod can maintain its curvature, leading to better correction or maintenance of TK after anatomical spinal correction surgery than the notched rod.

The diameter and material of rods play significant roles in deformity surgery and its maintenance over time^22,23^. Using the same titanium (φ 6.0 mm) and CoCr alloy rods (φ 5.5 mm) used in the present study, we previously demonstrated that the yield load of the manually bent CoCr alloy rods was almost identical to that of the manually bent titanium alloy rods, but the titanium alloy straight rods showed a significantly higher yield load than the straight CoCr alloy rods^14^. In addition, the previous study demonstrated that the notch-free, CoCr rods had a significantly higher ultimate load than the notched CoCr rods while having the same stiffness, indicating that the notch-free, curved CoCr alloy rod will likely maintain its shape compared with the notched rod^14^. Although the present study used different rod diameters and materials in the notched and notch-free rods, respectively, the results can be mainly explained based on the mechanical property induced by the notches within the rods. Notches result in local stress that surpasses the yield strength of the material, leading to plastic deformation^14,24,25^. The other possible mechanism of the observed better control in sagittal plain may be explained by inherent material property. Titanium alloy rods have a low work hardening ability while CoCr alloy rods have high work hardening ability and shape retention strength^14^. Although the two rods can yield at similar points, the curve to get there are different given different modulus. Thus, when yielded during the bending process, this will be stain hardened, and the when attempted to yield again (spring back) the yielding values will be higher for the stiffer material^14,25^.

When stress concentrations are introduced in the form of notches within rods, they play a considerable role in fatigue because rod fracture mainly occurs at the points of increased stress^14,24–26^. Rod contouring with a French bender significantly decreased the fatigue strength of both titanium and CoCr alloy rods^14,24,25^. In the current study, there was no implant breakage after surgery in either group; however, the notch-free rods developed for an adult spinal deformity would be more durable than the conventional notched rod.

Although it is unclear whether notch-free rods were supplied, industrially supplied pre-bent rods based on patient-specific data were reported to be effective in treating AIS patients^27^. Solla et al.^27^ treated 37 AIS patients with patient-specific rods that were pre-bent according to a protocol based on pelvic incidence. The rod contouring angles were determined using the pelvic incidence criteria (25° to 40° for the rod on the convex side and the same value plus 10° for the concave side), and CoCr rods were ordered before the date of surgery^27^. Although the postoperative overall TK was reported to be normal^27^, issues regarding the reconstruction of the anatomical TK remain. In addition, Solla et al.^27^ suggested that overbending the concave rod by ≥ 10° should be continued for patients with hypokyphosis because loss of sagittal correction related to rod flattening during correction is unavoidable.

In our surgical strategy, two rods were bent identically without reference to the preoperative spinal alignment^9^. This strategy is based on previous reports^11,13,28–30^. To overcome further lordosis of the thoracic spine after pedicle screw placements in thoracic AIS patients, we developed a surgical procedure wherein two rods are rotated simultaneously to correct the scoliosis while the TK is maintained or improved^4,9,11^. We previously evaluated changes in rod contour from before implantation to after this surgical correction, suggesting that the multilevel facetectomy and/or screw density at the concave side reduce the rod deformation^13^. Mobilization of the spine by releasing the facet joint is more important than using a rigid implant^9,31^. The present results indicate that neither a customized patient-specific rod nor overbending the concave rod is necessary for AIS surgery. Rod flattening is unavoidable, but the present results showed that the rod deformation angle was significantly lower in the notch-free rods than the notched rods in patients with preoperative hypokyphosis.

Since thoracic AIS patients have a preexistent hypokyphosis of the thoracic spine and the rods tend to flatten after surgery in these patients, anatomical rods have been developed for thoracic AIS patients. The rods can be particularly suitable for Lenke 1AR, in whom the apex of scoliosis is located at a lower thoracic spine^9^. However, we currently use these rods for other Lenke 2–6 curves except for Lenke 5 curve. Eleven rods are regarded as appropriate for pre-bent rods^15^. Although the configurations were obtained from Japanese patients, the algorithm to identify anatomical rod configurations can be applied for other races^15^. The algorithm to produce a pre-bent rod can also be utilized for Lenke 5 curve since the initial configuration leads to a certain sagittal alignment of the spine^15^. Another expected advantage of pre-bent rods is decreased operative time; however, the operative time was not significantly different between the groups in the present study.

There are limitations we must address. First, patient clinical outcome scores were not obtained since this study aimed to compare initial curve correction, as Gehrchen et al.^32^ compared the initial curve correction within 1 week after surgery using either circular rods or beam-like rods. The fact that notch-free pre-bent rods lead to improved sagittal control is purely a mechanical consideration. Although the notching within the rod could play a considerable role in fatigue performance, this would be relevant only if fusion did not occur after immediate correction. However, we previously reported that the average total Scoliosis Research Society questionnaire score significantly increased from 3.5 preoperatively to 4.5 at 2-year follow-up in the anatomical spinal reconstruction using manually bent rods^9^. In addition, the present preoperative and postoperative radiographic measurements in the manually bent rod group were almost identical to those of the previous study^9^. Second, the present study used different rod diameters and materials in the notched and notch-free rods, respectively. Few studies have evaluated mechanical properties of the curved rod^14,21^. In addition, to our knowledge, except for our previous study^14^, no studies have considered notch-free curved rods. As mentioned above, static four-point bending tests demonstrated similar load–displacement curves between the manually bent titanium 6.0 mm and CoCr 5.5 mm rods^14^. Based on the previous mechanical results^14^ and ethical reasons, we conducted the present study to compare the clinical results using manually bent titanium 6.0 mm rod and newly developed, notch-free curved CoCr 5.5 mm rod instead of manually bent CoCr 5.5 mm rods. However, even if similar yield and ultimate load were found for the manually bent titanium 6.0 mm and CoCr 5.5 mm rods, they share different stress–strain profiles to change the shape of the rod. In addition, both techniques used different screw hardware.

In conclusion, patients with notch-free, pre-bent rods had a significantly higher postoperative TK than patients with conventional, manually bent, notched rods after anatomical spinal correction surgery. These results suggest the potential benefits of anatomically designed notch-free, pre-bent rods over conventional, manually bent rods.

## Data Availability

The data that support the findings of this study are available from the corresponding author on reasonable request.
